# Exploring social‐emotional learning, school climate, and social network analysis

**DOI:** 10.1002/jcop.22881

**Published:** 2022-05-31

**Authors:** Maria Xu, Marisa MacDonnell, Angela Wang, Maurice J. Elias

**Affiliations:** ^1^ Psychology Department Rutgers, The State University of New Jersey Piscataway New Jersey USA

**Keywords:** character development, classrooms, peer leadership, peer relationships, school climate, social‐emotional learning, social network analysis

## Abstract

There is currently limited research on student peer leadership in the social‐emotional literature. This paper used exploratory methods of social network analysis to understand the structure of school peer relationships, peer leadership, and school climate. Self‐report measures of perceptions of peer leadership and climate were given to students during the 2016–2017 school year. Data collected from a peer leadership survey were used to calculate closeness and indegree centrality values. The results showed that student Ambassadors have higher peer nominated leadership scores compared to non‐Ambassador controls and the rest of the school. Additionally, Ambassadors did not demonstrate a change in centrality scores, non‐Ambassador students increased in centrality scores, and school climate was not correlated with the leadership centrality score. Results suggest that influence spreads, and that good leadership may be emulated among students, leading to a diffusion effect. This supports the need for good leaders in schools. Additionally, climate may not be associated with leadership centrality scores due to the length of the intervention. Future studies should look toward behavioral data to unravel what comprises positive and negative influences in Social‐Emotional and Character Development interventions.

## 1 INTRODUCTION

Hope, optimism, and self‐esteem have been found to be connected to better subjective well‐being, stronger family attachments and social support networks, and a greater capacity to make changes in the community and the self (Hamme Peterson et al., 2010). When youth live in poverty, they are more likely to feel hopeless, a sense of despair (Hamme Peterson et al., 2010), and to report higher levels of stress, compared to their peers in better socioeconomic circumstances (Vacek et al., 2010). Lack of resources, high quality education, safety, and positive pathways lead to greater likelihood of maladaptive behaviors, premature sexual behaviors, drug‐use, behavioral problems, delinquency, depression, and academic failure (Hamme Peterson et al., 2010; Vacek et al., 2010).

Interventions that focus on fostering self‐esteem and control over their environments can help counteract the negative effects of their circumstances (Berg et al., 2009; Foster‐Fishman et al., 2010; Ozer & Wright, 2012). For example, Youth Action Participatory Research programs can help encourage civic engagement, motivate traditionally disenfranchised students to act, teach valuable academic skills, help students collaborate with adults, and connect students with colleges and other institutions (Rubin & Jones, 2007). By giving students new responsibilities, an opportunity for leadership, and the tools to foster change, students can be inspired to lead their communities toward greater change and inspire others to do the same (Powers & Allaman, 2012).

The empowerment of youth may be helped by the selection of peer‐elected leaders who may influence the norms of their environments, including classroom settings. For youth who have had limited leadership opportunities, the possibility and actuality of serving in leadership roles can be a powerful boost to self‐efficacy and self‐concept. Consistent research has shown that problem behaviors may be impacted by peer influences (Cairns et al., 1988; Dishion et al., 1997; Ennett et al., 2006; Osgood et al., 2013). Research on bullying by Dijkstra et al. (2008) demonstrated that when popular students participated in bullying, bullying became more acceptable among students. Similarly, Jackson et al. (2015) found that students who exercise a type of aggression that “fit” the aggression norms of the classroom (relational vs. overt) are more liked while those that do not “fit” the aggression norms are less socially preferred. Studies by Dijkstra et al. (2008) and Jackson et al. (2015) suggest the importance of norms established in the classroom, the creation of norms based on social influence, and the possibility of targeting popular students' behavior to change the environment of the classroom overall. This includes students occupying leadership roles.

Social‐Emotional and Character Development (SECD) curricula can encourage student leadership and student empowerment as a method to promote social‐emotional learning (SEL) skills, foster character virtues, and establish positive norms (Linsky et al., 2018). There is little literature on the impact of SECD curricula that encourage student leadership, particularly peer‐elected student leadership, and their effectiveness in carrying out the goals of SECD for classrooms and the students in them.

### 1.1 An introduction to SECD and the role of student Ambassadors

In SECD, curricula typically focus on the five core competencies defined by the Collaborative for Academic, Social, and Emotional Learning (CASEL): self‐awareness, self‐management, social awareness, relationship skills, and responsible decision (CASEL, n.d.). The MOSAIC curriculum is a SECD approach suited for social network analysis (SNA). MOSAIC was created to help students understand and deal with emotions in a constructive manner, achieve positive goals, empathize with others, create, and maintain positive relationships, and encourage responsible decision making (Hatchimonji et al., 2017). MOSAIC used student leaders called Ambassadors to help carry out the curriculum. Peer‐elected leaders may help encourage healthy behaviors and discourage unhealthy behaviors by taking advantage of their status as opinion leaders (Starkey et al., 2009).

Student Ambassadors are students that are peer‐elected to serve in leadership roles in projects intended to have positive impacts on school and the greater community as a part of the MOSAIC used in this study. Ambassadors are trained on SEL skills such as relationship skills and encouraged to develop character virtues such as compassion to carry out visible positive community building. By exercising these skills in leadership positions, they are modeling to other students how to achieve a positive impact. This selection and training of already known and influential students were a part of a social network intervention embedded in a wider SECD intervention. In this study, the purpose of student peer leaders was to capitalize on already established peer influence to diffuse SEL skills and character virtues.

While very few Social Emotional Learning curricula use peer leaders to carry out their goals, the concept of using influential individuals, also known as opinion leaders, to diffuse information is not new. Hoy and Smith (2007) laid out 10 principles that leaders can foster to develop influence. The principle of colleagueship is pertinent as student Ambassadors were chosen based on their peers, so students are more likely to follow the lead of those who they already respect enough to elect to the position. The principles of expertise, trust, fairness, self‐efficacy, and optimism are principles that can be developed through the monthly training that Ambassadors take part in to learn how to carry out discussions in a sensitive and empathetic manner. Diffusion of information is also studied in other fields, such as climate policy (Luthfia & Alkhajar, 2020), video game settings (Z. Wang et al., 2020), or social media (Zhang et al., 2015). Many of these studies focus, however, more on simulation and mathematical models (Liu & Liu, 2018) instead of examining real world scenarios. What a diffusion process may look like in the context of our intervention would be that Ambassadors visibly become better at exercising their skills and virtues and do it more often over the year, they will also grow in their sphere of influence as more individuals see how they are positively impacted by their leadership. As more individuals look to their Ambassadors as good leaders, they may also emulate them in behavior and motivation, leading to the development of “secondary leaders” that will further influence others in a cascading effect.

An effective diffusion effect, therefore, would increase influence among Ambassadors relative to their peers, but it would also show a general (but significantly smaller) increase in cross‐influence among all students as they emulate their Ambassadors and set examples for others. If such a diffusion effect existed then it would have an impact on school climate, particularly on groups that have the greatest diffusion effect, because of the relationship between SECD and school climate improvement (Elias et al, 2010; Snyder et al., 2011). School climate, as defined by the National School Climate center, is the “quality and character of school life” and is “based on patterns of students', parents', and school personnel's experience of school life and reflects norms, goals, values, interpersonal relationships, teaching and learning practices, and organizational structures” (School Climate—National School Climate Center, 2022). School climate is important because of its ability to mitigate the negative effects of socioeconomic status differences (Berkowitz et al., 2016), prevent risks, promote health, student learning and academic achievement, increase student graduation rates, and increase teacher retention (Thapa et al., 2013). Although there is research about the influence of negative peer influence and school climate (Stewart, 2003; M. T. Wang & Dishion, 2011), there is little to no research on positive peer influence and school climate. A main focus of this paper is climate and peer leadership due to the numerous benefits that a positive school climate offers. Prior research demonstrating peer connectedness as a mediator of school climate (Loukas et al., 2006) suggests that peers likely play an influential role in students' perceptions of their school climate.

### 1.2 Ambassador training

Ambassadors, elected by their peers, were first introduced to their role through a contract and pledge. A discussion with a Q&A session was led by a respected school leader such as the school principal in the first meeting to clarify issues. Ambassadors met twice every month starting from November to February to work on developing an action plan with their classmates through discussions and presenting an action plan to change an aspect of their school as a part of their role as student leaders. From March to June, Ambassadors met with their teachers to carry out their action plan and to prepare for a showcase in June.

### 1.3 What is SNA and why use it?

SNA is an analytical process that can be used to help better understand how individuals fit in the context of their relationships with others (Powell & Hopkins, 2015). The evaluation of influence can be difficult to capture, but SNA is one potential way of investigating influence. Combined with nomination questions that ask students who they believe is a good leader, SNA can evaluate who students look toward for potential emulation, or in other words, who is influential. In SNA, respondents are often asked to nominate others based on their relationships, such as friendships, and this information can be converted into either a matrix or a list.

**Figure 1:**
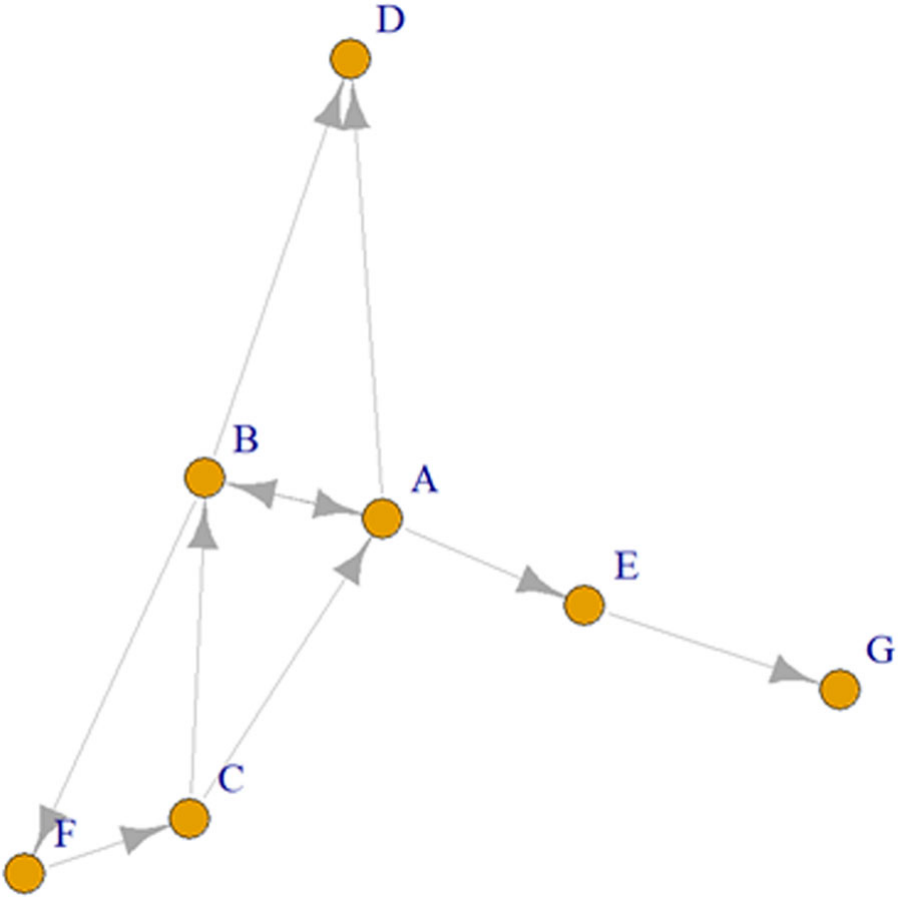
Example visualization of a social network

In a list form, this would be called an edge list in which the nominator is listed in the first column and the nominee is listed in the second column. This data format can then be used to create a visual representation of a graph. Figure 1 represents an example of a visualization of a social network graph. In a social network graph, a node represents an individual, such as a student that exists in a network. The circles in Figure 1 are nodes visually representing individuals. An edge represents a relationship between one node to another node. The arrows in Figure 1 are edges visually representing relationships. These edges can be weighted or unweighted (weighted edges have a value attached to the edge such as a 1 or 4). The sample graph in Figure 1 is unweighted. Some relationships are reciprocated while some are not, and this can be distinguished because this graph is directed. Graphs can be undirected or directed. Undirected edges mean that the relationship goes in both directions and is typically visually represented by lines without arrowheads. Directed edges indicate a unidirectional tie and are typically represented with a directional arrowhead.

Beyond visualizing data, converting the data into a matrix or a list is also useful because centrality can then be calculated. Visualizing data is not required for calculating centrality, but it is useful for identifying patterns. Centrality is a mathematical value calculated that can be used to model social influence by identifying individuals who are at critical positions in a network. An example of how centrality can be used would be to identify the node with the most amount of influence in controlling the flow of resources. The term centrality is a general term, and there are many different measures of centrality theorized to be able to identify individuals that hold a central position or position of influence, but there are two measures of centrality that are important for studies in SNA that this study will focus on: *Closeness centrality* and Ind*egree centrality*. *Closeness centrality* is the sum of a node's shortest distances from itself to all other nodes. Closeness centrality assumes that information always takes the shortest path along these edges, and it is often used to study flow of information in the most efficient manner (Borgatti, 2005). Individuals that are closer to others receive and send information faster. *Harmonic centrality* is a type of Closeness centrality calculated by the inverse summation of a node's shortest distance to all other nodes that is usable for disconnected graphs (Opsahl et al., 2010); however, we will refer to our Harmonic centrality values as Closeness centrality for simplicity. Our Closeness centrality values are calculated based on incoming nominations. *Indegree centrality* is the literal count of how many people nominated an individual and can be used as a measure of a direct influence (Borgatti, 2005).

Centrality values are important because centrality values can be used to identify the amount of influence an individual has relative to the whole group, and, therefore, centrality values can be used to identify popular students in large groups that impact norms and expand upon the work by Dijkstra et al. (2008) and Jackson et al. (2015) on the power of popular students in shaping social norms. Identifying influential power would allow interventions to monitor the effectiveness of interventions by tracking social influence of key individuals. PROmoting School‐community‐university Partnerships to Enhance Resilience (PROSPER), a program that has consistently helped reduce substance use. Osgood et al. (2013) showed that after the intervention, the centrality of antisocial students was reduced, and the centrality was refocused toward nonsubstance using students. Combining measures of centrality and questionnaires that ask for influence nominations can also help identify influential individuals in schools for interventions (Bevelander et al., 2018; Starkey et al., 2009). MyMovez is an example of a multidisciplinary health project carried out over 3 years (2016–2018) that used measures of centrality and different nomination questions, in contrast to friendship ties, to understand the impact of adolescence peer influence in a social network‐oriented health program (Bevelander et al., 2018).

### 1.4 Why Closeness centrality and Indegree centrality?

As stated above, *Closeness centrality* can be seen as a representation of flow of information. The closer a node is to every other node, the faster it can receive or send out information. When using questions that ask for nominations based on characteristics, it could be that Closeness centrality measures a specific type of information: peer influence. This sort of information would not be explicit, like emails sent, but would be a more indirect flow of information to peers about how to act and behave. The behavior of popular peers impacts social norms (Dijkstra et al., 2008), and the power of peer influence can reinforce positive prosocial behaviors, such as academic achievement, or reinforce negative behaviors, such as homophobic insults (Birkett & Espelage, 2014; Rambaran et al., 2016; van Hoorn et al., 2014). Friend groups and peer interaction can impact behavior, and peer relationships can be a source of information for an adolescent about what is acceptable (Jackson et al., 2015; Lapinski & Rimal, 2005). If nomination questions ask for nominations based on positive or prosocial characters (e.g., Who do you think is forgiving?), these questions could give us insight to who may be able to impact the greatest number of students.

*Indegree Centrality* is a simple count of how many individuals nominated a specific individual. This relationship can be outgoing, the person nominated other people in a survey, or incoming, the person was nominated by others. Instead of Degree centrality, which is based on how many ties there are between individuals, regardless of whether it is outgoing or incoming, Indegree centrality would be more appropriate because of the nature of the questions asked to collect social network data (see Section 2 for greater detail). The higher the Indegree value, the greater direct influence an individual would have on others.

It may be important to examine both Closeness centrality and Indegree centrality together. For instance, Rulison et al. (2014) showed that degree centrality negatively predicted diffusion of the PROSPER intervention. They reasoned that with high levels of degree centrality, the more centralized a social network is. In such a highly centralized network, information may be trapped within a small group. Gentina et al. (2015) showed that students with high Closeness centrality had greater ethical predispositions while students with high Degree centrality had lower ethical predispositions and higher likelihood of risky behaviors. This may be due to students with more direct ties perhaps feeling stronger peer pressure to conform to the group's specific norms while those with higher Closeness centrality may have felt the need to consider the behavior and norms of the classroom. Regarding the calculations themselves, Valente et al. (2008) showed that Indegree centrality is moderately correlated with Closeness centrality. The moderate correlation makes sense because more direct ties means closer distances between one node and other nodes, but if there is a tight connected network within a grander network, Closeness centrality values show that the person, although close to a small group of people directly, is not relatively close to all other nodes after all.

While direct influence may be desirable, Hill and Dunbar (2003) proposed that there is theoretically a max limit on the total number of quality friendships a person can have based on cognitive limits. This number has been estimated to be between 150 and 200 and analyses of social media interactions support the existence of this upper limit; after 150–200 relationships, the quality of relationship declines and cannot be maintained without hurting the quality of relationships with another (Gonçalves et al., 2011). Closeness centrality values paired with indegree values are important when evaluating a population greater than 200, such as a school, because of the limit of each individual to have quality relationships. Since students are unlikely to have over 200 quality friendships, they are unlikely to have considerable influence on everyone in the school. An influencer would have to depend on indirect influence. An ideal influencer, would therefore, have both high Indegree centrality, indicating close ties with a small network of friends and high Closeness centrality, which may indicate connections to the greater school and indirect influence outside of a small network.

### 1.5 Current study

Much of the research on SNA has focused on undirected friendship ties. This study looked at directed nomination ties with questions that specifically probe on character traits important to SEL and character development. This study is unique in that it uses nomination‐type questions to configure the networks.

**Figure 2:**
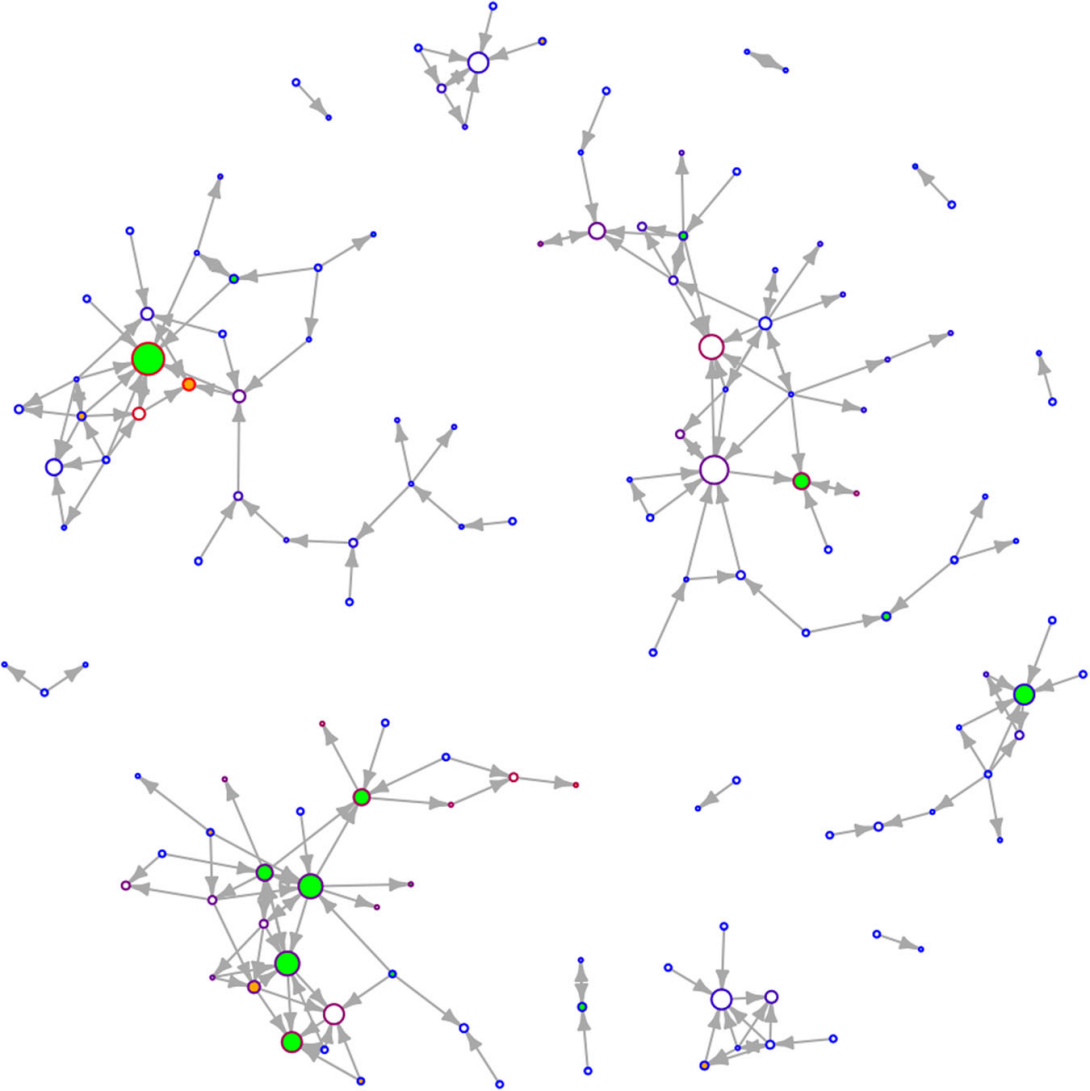
Good leader network of valid responses Fall 2016

**Figure 3:**
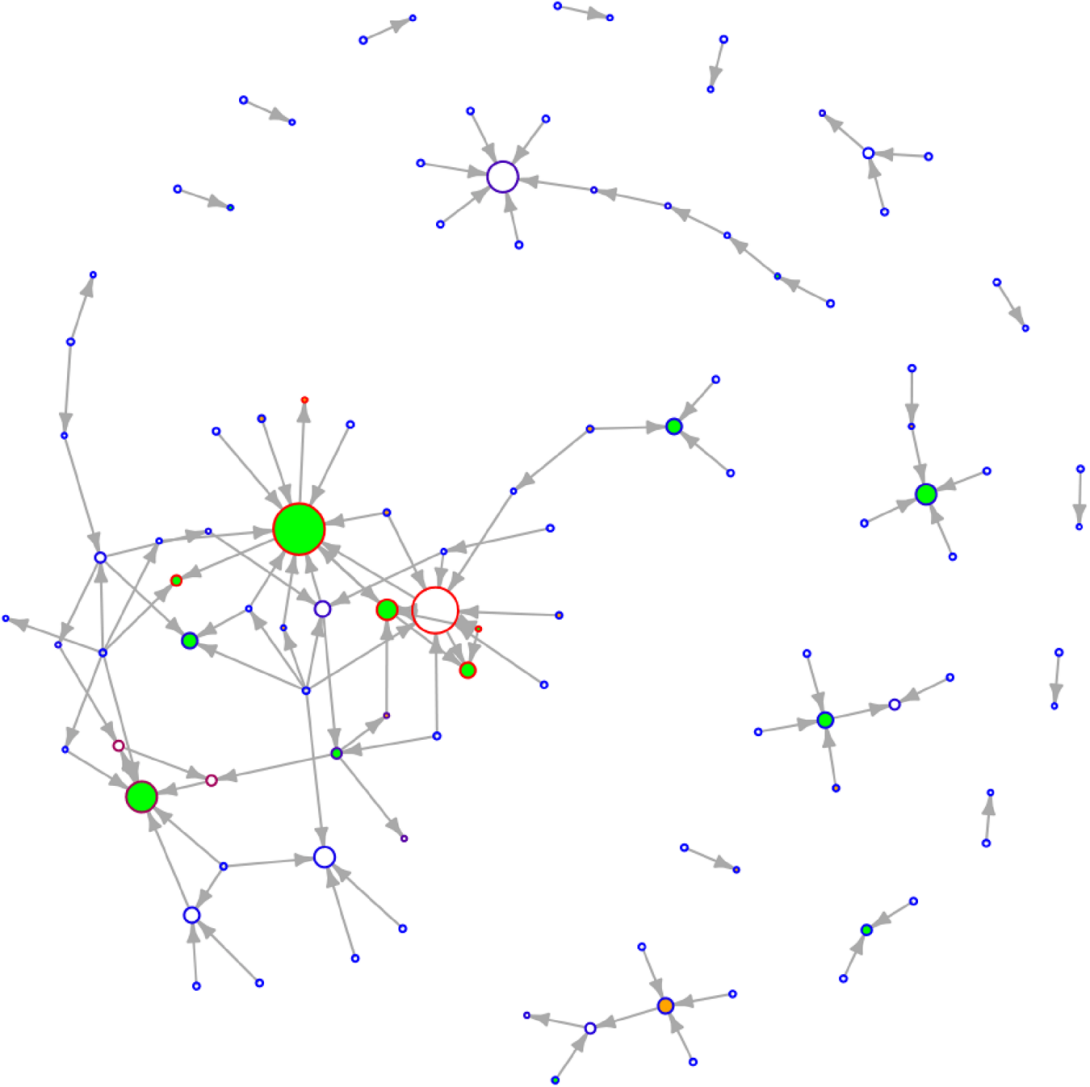
Good leader network of valid responses Spring 2017

The current study explored the relationship between climate ratings, student Ambassadors, and their network structures. The four major research questions are as follows:

1. Do Ambassadors increase in influence?
2. Do all students increase in influence?
3. Do peer leaders have more influence than their peers at both the beginning of the intervention and at the end?
4. Is there a relationship between influence and perceived school climate?

Our hypotheses to answer these questions are as follows:

Hypothesis 1 Peer leaders will increase in Closeness and Indegree centrality.

Hypothesis 2 Students overall will increase in Closeness and Indegree centrality.

Hypothesis 3 Peer leaders will have greater centrality values than students overall at the end of the year.

Hypothesis 4 Beginning of the year climate scores correlates with centrality scores.

Hypothesis 5 End of the year climate scores correlates with centrality scores.

## 2 METHODS

### 2.1 Design

This study is an exploratory case study and is meant to explore the uses of SNA in a SECD school context. We use descriptive statistics to summarize school climate and demographic characteristics. Student Ambassadors were matched with controls on demographic variables including race, gender, grade, and socioeconomic status.

### 2.2 Participant demographics

#### 2.2.1 Whole school demographics

The participants' data came from one urban New Jersey School District in the 2016–2017 school year. The data came from students in grades 6, 7, and 8. Each school had students that were elected as student Ambassadors at different points of the year. Any student that was an Ambassador at any point of the year was counted in as an Ambassador for the whole year. In 2016–2017, there were *n* = 312 total students in the school that stayed for the whole school year. 94.9% of students were of minority status, 47.3% were female, 85.8% were eligible for free or reduced lunch, and 27% were in special education. Out of all students, 125 were in 6th grade (40.06%), 91 were in 7th grade (29.17%), and 95 were in 8th grade (30.45%). Please see Table 1 for full information. Each Ambassador was matched with two other students based on race, grade, and gender. Two controls were chosen to reduce idiosyncrasy (Cook & Campbell, 1979). Students in the school who did not complete a survey were added onto the data set with 0 counts inputted for all questions. Please refer to Supporting Information: Tables A1–A4 for additional demographic info.

**Table 1:** Demographics of whole school, Ambassadors, and matched controls

	*N*	Female	Minority	Special education	Grade 6	Grade 7	Grade 8
Whole school	312	47.30%	94.90%	27%	40.06%	29.17%	30.45%
Ambassador	20	65%	95%	15%	20%	20%	60%
Control	38	68.42%	97.37%	34.21%	21.05%	26.32%	52.63%

#### 2.2.2 Ambassador and matched controls demographics

Two controls were chosen for each Ambassador to reduce idiosyncrasy when possible. In the 2016–2017 school year, 20 students were Ambassadors, 38 students were matched with the 20 Ambassadors. Among the Ambassadors, 95% of Ambassadors were of minority status, and 97.37% of matched controls were of minority status. For gender, 65% of Ambassadors were female, and 68.42% of matched controls were female. For eligibility, 80% of Ambassadors were eligible for free or reduced lunch, and 81.58% of matched controls were eligible for free or reduced lunch. For special education, 15% of Ambassadors were in special education, and 34.21% of matched controls were in special education. Of the Ambassadors, 4 were in 6th grade (20%), 4 were in 7th grade (20%), and 12 were in 8th grade (60%). Of the matched controls, 8 were in 6th grade (21.05%), 10 were in 7th grade (26.32%), and 20 were in 8th grade (52.63%).

### 2.3 Procedures

Students were asked to complete self‐report nomination surveys with six classroom level questions and six school level questions, given out in October and May. Surveys were completed during their standard advisory classes as activities. Parents gave permission to access student records on demographic characteristics and school performance data. Gender, ethnicity, grade‐level information was collected from school records. Data of students' perception of school climate were collected as a part of larger surveys given to the students. Students were not aware of any connection between the two survey procedures.

### 2.4 Measures

#### 2.4.1 MOSAIC leadership survey

Students were asked to nominate students within the school for six qualities that defined key elements of youth leadership: Making the Community Better, Being Compassionate, Being a Good Communicator, Being a Source of Good Problem Solving Advice, and Forgiveness. In addition, students were asked to nominate those in school they consider to be a Good Leader. They were given the option to nominate as many students as they could. The full list of questions and their abbreviations are listed in Table 2. For the purposes of this study, we only used Good Leader for our SNA because responses to this question correlated highly with responses to other qualities; factor analysis showed that all six attributes fell on a single dimension that could be defined as “Good Leader.”

**Table 2:** MOSAIC leadership survey questions

Variable name	Question
Good Leader	Who, in your whole school, do you think is a good leader?
Community Better	Who, in your whole school, wants to make your school and community better?
Compassionate	Who, in your whole school, is compassionate and shows concern for others?
Communication	Who, in your whole school, communicates well with others?
Problem Solving	Who, in your whole school, is helpful in solving a problem or getting something important done?
Forgiveness	Who, in your whole school, forgives others easily and does not hold grudges?

Response rates for the leadership survey were calculated based on the total number of entries received and recorded divided by the number of students at each time point. This includes entries that may be incomplete or left empty. The percentage of students who returned a valid response, the response rate, for those who completed Fall 2016 was 156/312 (50%) and Spring 2017 was 122/312 (39.10%). Special education students were included in the total count. Climate analyses were done on valid responses only.

#### 2.4.2 Climate survey

The climate survey was taken from the School as a Caring Community Profile‐ II, Student Form (SCCP‐II; Lickona & Davidson, 2004). The climate survey includes Likert‐type questions in which students rated their agreement to statements about school climate on a 5‐point scale (Disagree A LOT! = 1 to Agree A LOT! = 5). Students were asked to rate their perception of school climate on a total of 19 items in October and May. Of these 19 items, 13 items were categorized by the following subscales: Student Respect, Friendship and Belonging, Students' Shaping their Environment, Support and Care By and For Staff (Table 3). Six questions were miscellaneous and uncategorized. This study used the 13 items categorized into subscales.

**Table 3:** MOSAIC climate survey

Subscale	Question Content
Student Respect	Students treat classmates with respect. That means they are polite, think about others' feelings when with them, and don't say bad things to them.
	Students help each other, even if they are not friends.
	Students try to get other students to follow school rules.
Friendship and Belonging	Students work well together.
	Students help new students feel accepted.
	Students are willing to forgive each other. When you “forgive,” you are telling someone that you are not angry with them any more.
Students' Shaping their Environment	When students do something hurtful, they try to make up for it.
	Students resolve conflicts without fighting, insults, or threats. That means when students are upset with others or disagree, they will find a way to deal with it without fighting, insulting, or threatening others.
	Students are involved in helping to solve school problems.
Support and Care by and for Staff	Students can talk to their teachers about problems that are bothering them.
	Teachers go out of their way to help students who need extra help.
	In this school you can count on adults to try to make sure students are safe.
	Teachers in this school like to come here.

### 2.5 SNA data analysis plan

Nominations from the student survey were converted into indegree files using R. Self‐nominations were removed. Closeness centrality was then calculated using R and the tnet package created by Opsahl et al. (2010). The tnet package, an additional and downloadable toolbox of usable code, contains R functions that can be used to calculate Closeness centrality with graphs that have “holes” in them. The holes occur because of isolates or students that were not nominated but still exist in the network separate from everyone else. The normalized Closeness centrality values were calculated for each question and by time point.

## 3 RESULTS

Preliminary analyses were done to assess if there were differences across climate scores by gender, grade level, eligibility, and race. Independent *t*‐tests were done on both time points for gender (Table 4). There were significant differences between gender on all measures. Additionally, analysis of variances (ANOVAs) were run to assess for group differences between grade level, eligibility, and race. For Spring 2017 only, ANOVAs results showed that there were significant differences in climate scores between grade level for Student Respect *F*(2, 307) = 4.018, *p* = .019), Friendship and Belonging *F*(2, 307) = 4.087, *p* = .018, Students Shaping their Environments *F*(2, 307) = 3.906, *p* = .021, and Support and Care by and For Staff *F*(2, 307) = 4.908, *p* = .008. Post hoc tests (Tukey's HSD) found that 7th graders rated climate higher than 6th and 8th graders on all subscales. No other groups were significantly different.

**Table 4:** Climate scores differences between gender

Semester	Item	Female mean and SD	Male mean and SD	*t* Value	*p* Value
Fall 2016	Student Respect	*M* = 1.27, SD = 1.687	*M* = 0.67, SD = 1.367	*t*(309) = 3.463	.001
	Friendship and Belonging	*M* = 1.45, SD = 1.799	*M* = 0.77, SD = 1.520	*t*(309) = 3.623	.000
	Students Shaping their Environments	*M* = 1.23, SD = 1.637	*M* = 0.65, SD = 1.329	*t*(309) = 3.446	.001
	Support and Care by and for Staff	*M* = 1.53, SD = 1.923	*M* = 0.85, SD = 1.650	*t*(309) = 3.371	.001
Spring 2017	Student Respect	*M* = 0.55, SD = 1.268	*M* = 0.26, SD = 0.942	*t*(309) = 2.278	.023
	Friendship and Belonging	*M* = 0.59, SD = 1.344	*M* = 0.28, SD = 1.002	*t*(309) = 2.314	.021
	Students Shaping their Environments	*M* = 0.54, SD = 1.215	*M* = 0.27, SD = 0.935	*t*(309) = 2.220	.027
	Support and Care by and for Staff	*M* = 0.65, SD = 1.422	*M* = 0.28, SD = 0.981	*t*(309) = 2.680	.008

**Table 5:** Permutation paired *t*‐test comparing centrality across time points

Category	Centrality	Standardized *t*‐statistic	Difference in means	*p* Value
All students	Indegree	3.3699	8.2447	<.001
	Closeness	4.3477	13.5505	<.001
All students without ambassadors	Indegree	4.6326	7.9041	<.001
	Closeness	4.5706	15.8699	<.001
All students without ambassadors and controls	Indegree	4.3	7.9528	<.001
	Closeness	4.748	16.3661	<.001
Ambassadors only	Indegree	0.798	12.95	.4347
	Closeness	−0.55	−20.35	.5888

For the analyses, the nature of centrality measures breaks the rule of independence, so instead of normal paired *t*‐tests, permutation *t*‐tests using R's “MKinfer” package, were ran instead to compare the Fall 2016 scores and the Spring 2017 scores. The indegree and Closeness centrality scores of all students, all students without Ambassadors, and all students without Ambassadors and controls were compared across time points and there was a significant difference between the means at *p* < .001 (Table 5).

The indegree and Closeness centrality scores of Ambassadors only were compared and there was no significant difference. Regular permutation *t*‐tests were run to compare Ambassadors to controls at each time point. At every time point, there was a significant difference between Ambassadors and controls at *p* < .001 (Table 6).

Correlations were ran between centrality scores and climate scores, and there was no correlation between centrality scores and climate scores except a negative correlation between Climate question 2 (Student Respect) and Indegree centrality *r*(103) = −0.202, *p* < .05 and Closeness centrality *r*(103) = −0.231, *p* < .5 and a negative correlation between Climate question 13 (Support and Care By and For Staff) and Indegree centrality *r*(101)=−2.15, *p* < .05 and Closeness centrality *r*(101)=−0.269, *p* < .01. Please see Table 7 for full information.

## 4 DISCUSSION

This paper explored the relationship between peer leadership, climate, and the structural networks of students in a school with closely knit Ambassadors. Most studies evaluating SECD have not focused on the contextual level of analyses that SNA offers and even those few have focused on friendship ties. This study looked at nomination questions that are unique to SECD to understand how good leadership develops in schools. The networks created by this SNA are visualized in Figure 2 and Figure 3.This was created to help visualize what a nomination network looks like and what the results correspond to. A green filled‐in circle represents an Ambassador. An orange filled‐in circle represents a matched control. Larger circles indicate an higher indegree value while smaller circles indicate a lower indegree value. The more red a circle is, the higher the closeness centrality while the more blue a circle is, the lower the closeness centrality.In the primarily analyses, there were significant differences between grade levels on climate scores, and that students identifying as female rated the school climate higher on all climate scores. Further analyses identified that 7th graders rated school climate higher than 6th and 8th graders. In the main analyses, Ambassadors and other students had significantly different scores at both time points, that students overall, even when removing Ambassadors and controls, increased in centrality scores, but Ambassadors themselves did not increase in centrality scores, and that climate is not correlated with centrality scores but is rather negatively correlated in two instances during Spring 2017.

The gender difference in reporting of climate is consistent with prior research on the area. Koth et al. (2008) found that students identifying as female tended to rate climate scores higher than male students in their sample. White et al. (2014) also found that students identifying as female rated climate higher than students identifying as male using their Georgia Brief School Climate Inventory (GaBSCI). This may be the consequence of who students interact with and gender roles. Students identifying as female are socialized by their peers to be more likely to engage in prosocial behaviors, like listening, and less in aggressive behaviors (Witt, 2000). Differences in grade level may reflect a seniority effect. Students in 7th grade received 2 years of MOSAIC, in comparison to 6th graders who received only 1 year of MOSAIC, may have been more receptive to its effects because they started out younger than the 8th graders. Future research suggestions based on these results are elaborated upon below.

Hypotheses 1 and 2 were supported by the paired permutation *t*‐tests. It makes sense that Ambassadors and students would have significantly different scores at the beginning of the intervention as well as the end of the intervention because Ambassadors were chosen by their peers to be student leaders. To be chosen as a student leader suggests that they were already influential students. Their maintenance of scores suggests that one tenet of the diffusion effect occurred, however, the other tenet only partially occurred. Hypothesis 3 was not support by our results. Students over all did increase in influence as suggested by our theory of diffusion, but Ambassadors did not increase in influence overall. This may be due to a ceiling effect where student leaders could not have grown more in influence because they were already near the top of influence in the first place. The maintenance of higher influence suggests that even though they did not grow in influence, Ambassadors still maintained a critical role within the community even at the end of the year.

Hypotheses 4 and 5 were not support by our results, and the results suggested the opposite of our hypotheses (see Supporting Information: Table 5a in Appendix).

Students with high influence were less likely to see their fellow classmates helping others even if they were not friends. They were also more likely to rate teachers lower on wanting to be in school. This suggests that there might be a certain culture among high influencers that makes them less likely to see students helping others and teachers liking to be at the school. On one hand, this could be a more accurate portrayal of what students are feeling at the schools, but on the other hand, we did not look at Ambassadors only versus non‐Ambassadors or controls. It could be that the effect only stays true for high centrality non‐Ambassadors. This would then suggest the presence of negative influences in the school that could offset the positive influences of the Ambassadors. Dijkstra et al. (2008) and Jackson et al. (2015)'s research on antisocial behavior suggests that the power of negative influence can easily diffuse and impact others just as much as positive behavior. Similarly, Gentina et al. (2015) showed that students with high degree centrality had lower ethical predispositions and higher likelihood of risky behaviors than students with high Closeness centrality. Future studies should look at the complex interplay between positive and negative influences by identifying behaviors that represent prosocial versus antisocial virtues.

One of the major limitations of this study is that the measures of centrality may simply be an indicator of popularity. This would mean that the “positive” influence of the Ambassador is negative or neutral in many ways rather than positive in the way that SECD tries to promote. Dijkstra et al. (2013) found that low popularity students from a middle school sample try to associate with higher popularity students to increase their own popularity while higher popularity students try to keep distance from lower popularity students to prevent their own status from dropping. This sort of pattern of association may explain why Ambassadors did not increase in influence but other students did. The popular students that were nominated did their best to maintain their status from dropping while lower popularity students try to climb the ladder and make more ties with the popular and more influential students.

Additionally, it may be more difficult to disseminate positive influence comparatively in larger groups of students. Moldovan et al. (2017) found that opinion leaders are more effective in smaller groups and that group size is negatively associated with level of influence. Additionally, evidence from Rulison et al. (2014) showed us that high degree centrality is related to low diffusion of information. Considering how large the school is, it is possible that the positive influence may not have spread as much outside the confines of small‐knit clusters. This may also be complicated by time as we only looked at the influence of the intervention over 1 year rather than across years. There might be a difference in climate scores in a positive direction when looking at the intervention across years that could suggest a successful diffusion effect that generated an overall positive impact.

### 4.1 Suggestions for future research

Future studies should focus on exploring further the structural components of networks and the relationship to school climate through measures such as density. Additionally, future research should look at measures such as Katz centrality (Borgatti, 2005) to consider the declining nature of influence the further away a node is from another node. Future interventions should be informed by our findings; program developers should consider having Ambassadors work closely together to increase the odds that influence is spread out amongst the overall network. Hopefully, by encouraging Ambassadors to work together, younger Ambassadors will also learn how to be good leaders from older students that may be more influential or feel more comfortable in their roles such as the 7th graders who had higher climate scores than 8th and 6th graders. Careful attention should be placed on making sure influence should be centered around students with good leadership skills and prosocial behavior.

**Table 6:** Permutation *t*‐tests on centrality scores between Ambassadors and matched controls

Semester	Centrality	Ambassador mean	Matched control mean	*p* Value
F16	Indegree	0.009	0.001	.0001
S17	Indegree	0.007	0.001	.0001
F16	Closeness	0.014	0.003	.0004
S17	Closeness	0.016	0.002	.0002

Future studies should also consider examining whether the influence is positive through behavioral measures and additional reports from teachers and other faculty. This would help untangle why certain patterns occur in the data such as an Ambassador not increasing significantly in centrality scores. This, coupled with examination of specific groups of individuals with high climate scores, may illuminate who may be a positive force in the intervention, and who may be an influence counter to the goals of the intervention.

**Table 7:** Correlations between climate and centrality scores

Question	F16 Indegree	F16 Closeness	Question	S17 Indegree	S17 Closeness
F16 Climate 1			S17 Climate 1		
Pearson's correlation	−0.038	−0.067	Pearson's correlation	−0.151	−0.149
Sig. (two‐tailed)	0.619	0.388	Sig. (two‐tailed)	0.131	0.135
*N*	170	170	*N*	102	102
F16 Climate 2			S17 Climate 2		
Pearson's correlation	−0.045	−0.060	Pearson's correlation	−0.202*	−0.231*
Sig. (two‐tailed)	0.561	0.439	Sig. (two‐tailed)	0.039	0.018
*N*	169	169	*N*	104	104
F16 Climate 3			S17 Climate 3		
Pearson's correlation	0.030	0.018	Pearson's correlation	−0.171	−0.180
Sig. (two‐tailed)	0.699	0.817	Sig. (two‐tailed)	0.085	0.069
*N*	168	168	*N*	103	103
F16 Climate 4			S17 Climate 4		
Pearson's correlation	−0.008	−0.088	Pearson's correlation	−0.148	−0.153
Sig. (two‐tailed)	0.914	0.262	Sig. (two‐tailed)	0.132	0.121
*N*	165	165	*N*	104	104
F16 Climate 5			S17 Climate 5		
Pearson's correlation	−0.030	−0.039	Pearson's correlation	−0.093	−0.077
Sig. (two‐tailed)	0.697	0.617	Sig. (two‐tailed)	0.355	0.439
*N*	167	167	*N*	102	102
F16 Climate 6			S17 Climate 6		
Pearson's correlation	−0.041	−0.084	Pearson's correlation	−0.162	−0.181
Sig. (two‐tailed)	0.599	0.278	Sig. (two‐tailed)	0.101	0.067
*N*	170	170	*N*	104	104
F16 Climate 7			S17 Climate 7		
Pearson's correlation	0.074	0.030	Pearson's correlation	0.032	0.020
Sig. (two‐tailed)	0.343	0.704	Sig. (two‐tailed)	0.747	0.838
*N*	166	166	*N*	104	104
F16 Climate 8			S17 Climate 8		
Pearson's correlation	−0.102	−0.116	Pearson's correlation	−0.116	−0.120
Sig. (two‐tailed)	0.191	0.136	Sig. (two‐tailed)	0.245	0.227
*N*	166	166	*N*	103	103
F16 Climate 9			S17 Climate 9		
Pearson's correlation	−0.069	−0.109	Pearson's correlation	0.008	0.018
Sig. (two‐tailed)	0.379	0.162	Sig. (two‐tailed)	0.933	0.854
*N*	167	167	*N*	103	103
F16 Climate 10			S17 Climate 10		
Pearson's correlation	0.027	−0.026	Pearson's correlation	−0.054	−0.078
Sig. (two‐tailed)	0.731	0.736	Sig. (two‐tailed)	0.586	0.432
*N*	166	166	*N*	103	103
F16 Climate 11			S17 Climate 11		
Pearson's correlation	0.033	−0.013	Pearson's correlation	−0.131	−0.165
Sig. (two‐tailed)	0.678	0.864	Sig. (two‐tailed)	0.187	0.096
*N*	164	164	*N*	103	103
F16 Climate 12			S17 Climate 12		
Pearson's correlation	−0.106	−0.130	Pearson's correlation	−0.162	−0.165
Sig. (two‐tailed)	0.176	0.097	Sig. (two‐tailed)	0.101	0.096
*N*	165	165	*N*	103	103
F16 Climate 13			S17 Climate 13		
Pearson's correlation	−0.141	−0.151	Pearson's correlation	−0.215*	−0.269**
Sig. (two‐tailed)	0.071	0.053	Sig. (two‐tailed)	0.030	0.006
*N*	165	165	*N*	102	102

*p < 0.05; **p < 0.01.

Finally, future research should consider the impact of demographic variables on the outcome of the intervention. There may be gender differences that complicate the dissemination of SECD intervention, and if SNA can identify how these gender differences play out in influence spread, future intervention can then use this information to target specific individuals and groups to encourage the spread of the intervention. The same methods can be used to unravel why grade differences exist and what can be done to further encourage the diffusion of SECD skills and virtues.

### 1 PEER REVIEW

The peer review history for this article is available at https://publons.com/publon/10.1002/jcop.22881

## Supporting information

Supporting information.

## Data Availability

The data that support the findings of this study are available from the corresponding author upon reasonable request.
