# Supplementary material for: Exploring social‐emotional learning, school climate, and social network analysis
Source: J Community Psychol. 2022 May 31;51(1):84–102. doi: 10.1002/jcop.22881 (PMC10084104; doi:10.1002/jcop.22881)
Supplement: Supplementary file 1 — Supporting information. [file JCOP-51-84-s001.docx]

| **Table A1** | | | | | |
| --- | --- | --- | --- | --- | --- |
| Regular Students (Non-Ambassador and Non-Control) Centrality and Climate Descriptive Analyses | | | | | |
| Measure | Valid N | Minimum | Maximum | Mean | Std. Deviation |
| F16 Indegree | 254 | 0 | 22.51 | 1.5697 | 3.3915 |
| S17 Indegree | 254 | 0 | 28.94 | 0.7722 | 2.74474 |
| F16 Closeness | 254 | 0 | 32.69 | 2.8553 | 5.98668 |
| S17 Closeness | 254 | 0 | 47.19 | 1.2184 | 4.54632 |
| F16 Student Respect Q1 | 170 | 1 | 5 | 3.02 | 1.395 |
| F16 Student Respect Q2 | 169 | 1 | 5 | 3.03 | 1.462 |
| F16 Student Respect Q3 | 168 | 1 | 5 | 2.86 | 1.322 |
| F16 Friendship and Belonging Q1 | 165 | 1 | 5 | 3.08 | 1.384 |
| F16 Friendship and Belonging Q2 | 167 | 1 | 5 | 3.42 | 1.132 |
| F16 Friendship and Belonging Q3 | 170 | 1 | 5 | 3.68 | 1.209 |
| F16 Students' Shaping their Environment Q1 | 166 | 1 | 5 | 3.25 | 1.198 |
| F16 Students' Shaping their Environment Q2 | 166 | 1 | 5 | 2.75 | 1.421 |
| F16 Students' Shaping their Environment Q3 | 167 | 1 | 5 | 3.4 | 1.299 |
| F16 Support and Care By and For Staff Q1 | 166 | 1 | 5 | 3.61 | 1.338 |
| F16 Support and Care By and For Staff Q2 | 164 | 1 | 5 | 3.84 | 1.223 |
| F16 Support and Care By and For Staff Q3 | 165 | 1 | 5 | 3.56 | 1.111 |
| F16 Support and Care By and For Staff Q4 | 165 | 1 | 5 | 3.95 | 1.058 |
| S17 Student Respect Q1 | 102 | 1 | 5 | 3.43 | 1.316 |
| S17 Student Respect Q2 | 104 | 1 | 5 | 3.37 | 1.387 |
| S17 Student Respect Q3 | 103 | 1 | 5 | 2.91 | 1.307 |
| S17 Friendship and Belonging Q1 | 104 | 1 | 5 | 2.81 | 1.3 |
| S17 Friendship and Belonging Q2 | 102 | 1 | 5 | 3.02 | 1.35 |
| S17 Friendship and Belonging Q3 | 104 | 1 | 5 | 3.49 | 1.285 |
| S17 Students' Shaping their Environment Q1 | 104 | 1 | 5 | 3.33 | 1.194 |
| S17 Students' Shaping their Environment Q2 | 103 | 1 | 5 | 3.02 | 1.386 |
| S17 Students' Shaping their Environment Q3 | 103 | 1 | 5 | 2.77 | 1.173 |
| S17Support and Care By and For Staff Q1 | 103 | 1 | 5 | 3.2 | 1.158 |
| S17 Support and Care By and For Staff Q2 | 103 | 1 | 5 | 3.5 | 1.22 |
| S17 Support and Care By and For Staff Q3 | 103 | 1 | 5 | 3.21 | 1.242 |
| S17 Support and Care By and For Staff Q4 | 102 | 1 | 5 | 3.41 | 1.338 |

| **Table A2** | | | | | |
| --- | --- | --- | --- | --- | --- |
| Ambassadors Centrality and Climate Descriptive Analyses | | | | | |
| Measure | Valid N | Minimum | Maximum | Mean | Std. Deviation |
| F16 Indegree | 20 | 0 | 25.72 | 8.5209 | 7.82592 |
| S17 Indegree | 20 | 0 | 32.15 | 7.2347 | 8.00467 |
| F16 Closeness | 20 | 0 | 37.38 | 13.5117 | 12.47185 |
| S17 Closeness | 20 | 0 | 53.97 | 15.5483 | 17.1944 |
| F16 Student Respect Q1 | 20 | 1 | 5 | 2.55 | 1.276 |
| F16 Student Respect Q2 | 20 | 1 | 5 | 2.6 | 1.465 |
| F16 Student Respect Q3 | 20 | 1 | 5 | 2.5 | 1.1 |
| F16 Friendship and Belonging Q1 | 20 | 1 | 5 | 2.6 | 1.353 |
| F16 Friendship and Belonging Q2 | 20 | 1 | 4 | 3 | 0.973 |
| F16 Friendship and Belonging Q3 | 20 | 1 | 5 | 3.75 | 1.372 |
| F16 Students' Shaping their Environment Q1 | 20 | 1 | 5 | 3.2 | 1.196 |
| F16 Students' Shaping their Environment Q2 | 20 | 1 | 4 | 2.25 | 1.118 |
| F16 Students' Shaping their Environment Q3 | 20 | 1 | 5 | 2.8 | 1.196 |
| F16 Support and Care By and For Staff Q1 | 20 | 1 | 5 | 3.85 | 1.348 |
| F16 Support and Care By and For Staff Q2 | 20 | 1 | 5 | 3.85 | 1.089 |
| F16 Support and Care By and For Staff Q3 | 20 | 1 | 5 | 3 | 1.298 |
| F16 Support and Care By and For Staff Q4 | 20 | 1 | 5 | 3.55 | 1.356 |
| S17 Student Respect Q1 | 13 | 1 | 5 | 3.31 | 1.494 |
| S17 Student Respect Q2 | 13 | 1 | 5 | 3.46 | 1.45 |
| S17 Student Respect Q3 | 13 | 1 | 5 | 2.38 | 1.325 |
| S17 Friendship and Belonging Q1 | 13 | 1 | 5 | 2.62 | 1.261 |
| S17 Friendship and Belonging Q2 | 13 | 1 | 5 | 2.85 | 1.144 |
| S17 Friendship and Belonging Q3 | 13 | 2 | 5 | 3.15 | 0.987 |
| S17 Students' Shaping their Environment Q1 | 13 | 1 | 5 | 3.08 | 1.32 |
| S17 Students' Shaping their Environment Q2 | 13 | 1 | 5 | 2.69 | 1.182 |
| S17 Students' Shaping their Environment Q3 | 12 | 1 | 5 | 2.75 | 1.215 |
| S17Support and Care By and For Staff Q1 | 13 | 1 | 4 | 3 | 1.08 |
| S17 Support and Care By and For Staff Q2 | 13 | 1 | 5 | 3.62 | 1.044 |
| S17 Support and Care By and For Staff Q3 | 13 | 1 | 5 | 3.38 | 1.193 |
| S17 Support and Care By and For Staff Q4 | 13 | 1 | 5 | 3.31 | 1.182 |

| **Table A3** | | | | | |
| --- | --- | --- | --- | --- | --- |
| Controls Centrality and Climate Descriptive Analyses | | | | | |
| Measure | Valid N | Minimum | Maximum | Mean | Std. Deviation |
| F16 Indegree | 38 | 0 | 9.65 | 1.4385 | 2.66386 |
| S17 Indegree | 38 | 0 | 9.65 | 0.6769 | 1.85511 |
| F16 Closeness | 38 | 0 | 32.02 | 3.0132 | 6.75586 |
| S17 Closeness | 38 | 0 | 34.41 | 1.7572 | 6.06592 |
| F16 Student Respect Q1 | 19 | 1 | 5 | 3.05 | 1.353 |
| F16 Student Respect Q2 | 20 | 1 | 5 | 2.95 | 1.317 |
| F16 Student Respect Q3 | 20 | 1 | 5 | 3.45 | 1.638 |
| F16 Friendship and Belonging Q1 | 20 | 1 | 5 | 3.1 | 1.21 |
| F16 Friendship and Belonging Q2 | 20 | 1 | 5 | 3.3 | 1.261 |
| F16 Friendship and Belonging Q3 | 20 | 1 | 5 | 3.85 | 1.226 |
| F16 Students' Shaping their Environment Q1 | 20 | 1 | 5 | 3.15 | 1.268 |
| F16 Students' Shaping their Environment Q2 | 19 | 1 | 5 | 2.58 | 1.387 |
| F16 Students' Shaping their Environment Q3 | 19 | 1 | 5 | 3.05 | 1.268 |
| F16 Support and Care By and For Staff Q1 | 19 | 1 | 5 | 3.53 | 1.429 |
| F16 Support and Care By and For Staff Q2 | 19 | 2 | 5 | 4 | 1.106 |
| F16 Support and Care By and For Staff Q3 | 20 | 1 | 5 | 3.6 | 1.273 |
| F16 Support and Care By and For Staff Q4 | 20 | 1 | 5 | 3.3 | 1.593 |
| S17 Student Respect Q1 | 19 | 1 | 5 | 3.21 | 1.398 |
| S17 Student Respect Q2 | 19 | 1 | 5 | 2.79 | 1.357 |
| S17 Student Respect Q3 | 18 | 1 | 5 | 3.28 | 1.274 |
| S17 Friendship and Belonging Q1 | 16 | 1 | 4 | 2.38 | 1.31 |
| S17 Friendship and Belonging Q2 | 18 | 1 | 5 | 3.33 | 1.372 |
| S17 Friendship and Belonging Q3 | 17 | 1 | 5 | 3.59 | 1.228 |
| S17 Students' Shaping their Environment Q1 | 18 | 1 | 5 | 3.11 | 1.231 |
| S17 Students' Shaping their Environment Q2 | 19 | 1 | 5 | 3.42 | 1.346 |
| S17 Students' Shaping their Environment Q3 | 19 | 1 | 5 | 2.53 | 1.264 |
| S17Support and Care By and For Staff Q1 | 19 | 1 | 5 | 3.05 | 1.224 |
| S17 Support and Care By and For Staff Q2 | 19 | 1 | 5 | 3.84 | 0.958 |
| S17 Support and Care By and For Staff Q3 | 19 | 1 | 5 | 3.16 | 1.015 |
| S17 Support and Care By and For Staff Q4 | 19 | 1 | 5 | 3.53 | 1.219 |

| **Table A4** | | | | | | | | | | | | | |  | |
| --- | --- | --- | --- | --- | --- | --- | --- | --- | --- | --- | --- | --- | --- | --- | --- |
| Demographics by Student Condition | | | | | | | | | | | | | |  | |
| Race | Regular Student | Regular Student % | Ambassador | | Ambassador % | | Control | | Control % | | Total | | Total % | |  |
| American Indian | 1 | 0% | | 0 | | 0% | | 0 | | 0% | | 1 | | 0% | |
| Asian | 22 | 9% | | 0 | | 0% | | 0 | | 0% | | 22 | | 7% | |
| Black | 150 | 59% | | 7 | | 35% | | 14 | | 37% | | 171 | | 55% | |
| Hispanic | 59 | 23% | | 11 | | 55% | | 21 | | 55% | | 91 | | 29% | |
| Multiracial | 3 | 1% | | 0 | | 0% | | 0 | | 0% | | 3 | | 1% | |
| Pacific Islander | 4 | 2% | | 1 | | 5% | | 2 | | 5% | | 7 | | 2% | |
| White | 14 | 6% | | 1 | | 5% | | 1 | | 3% | | 16 | | 5% | |
| Gender | Regular Student | Regular Student % | | Ambassador | | Ambassador % | | Control | | Control % | | Total | | Total % | |
| Male | 145 | 57% | | 7 | | 35% | | 12 | | 32% | | 164 | | 53% | |
| Female | 108 | 43% | | 13 | | 65% | | 26 | | 68% | | 147 | | 47% | |
| Special Education | Regular Student | Regular Student % | | Ambassador | | Ambassador % | | Control | | Control % | | Total | | Total % | |
| No | 185 | 73% | | 17 | | 85% | | 25 | | 66% | | 227 | | 73% | |
| Yes | 68 | 27% | | 3 | | 15% | | 13 | | 34% | | 84 | | 27% | |
| Free or Reduced Lunch | Regular Student | Regular Student % | | Ambassador | | Ambassador % | | Control | | Control % | | Total | | Total % | |
| Paid | 33 | 13% | | 4 | | 20% | | 7 | | 18% | | 44 | | 14% | |
| Reduced | 26 | 10% | | 2 | | 10% | | 1 | | 3% | | 29 | | 9% | |
| Free | 194 | 77% | | 14 | | 70% | | 30 | | 79% | | 238 | | 77% | |

| **Table 5a** | | | | | | | |
| --- | --- | --- | --- | --- | --- | --- | --- |
| Correlations Between Climate and Centrality Scores | | | | | | | |
| Question |  | F16Indegree | F16Closeness | Question |  | S17Indegree | S17Closeness |
| F16 Climate 1 | Pearson Correlation | -0.038 | -0.067 | S17 Climate 1 | Pearson Correlation | -0.151 | -0.149 |
|  | Sig. (2-tailed) | 0.619 | 0.388 |  | Sig. (2-tailed) | 0.131 | 0.135 |
|  | N | 170 | 170 |  | N | 102 | 102 |
| F16 Climate 2 | Pearson Correlation | -0.045 | -0.060 | S17 Climate 2 | Pearson Correlation | -.202^*^ | -.231^*^ |
|  | Sig. (2-tailed) | 0.561 | 0.439 |  | Sig. (2-tailed) | 0.039 | 0.018 |
|  | N | 169 | 169 |  | N | 104 | 104 |
| F16 Climate 3 | Pearson Correlation | 0.030 | 0.018 | S17 Climate 3 | Pearson Correlation | -0.171 | -0.180 |
|  | Sig. (2-tailed) | 0.699 | 0.817 |  | Sig. (2-tailed) | 0.085 | 0.069 |
|  | N | 168 | 168 |  | N | 103 | 103 |
| F16 Climate 4 | Pearson Correlation | -0.008 | -0.088 | S17 Climate 4 | Pearson Correlation | -0.148 | -0.153 |
|  | Sig. (2-tailed) | 0.914 | 0.262 |  | Sig. (2-tailed) | 0.132 | 0.121 |
|  | N | 165 | 165 |  | N | 104 | 104 |
| F16 Climate 5 | Pearson Correlation | -0.030 | -0.039 | S17 Climate 5 | Pearson Correlation | -0.093 | -0.077 |
|  | Sig. (2-tailed) | 0.697 | 0.617 |  | Sig. (2-tailed) | 0.355 | 0.439 |
|  | N | 167 | 167 |  | N | 102 | 102 |
| F16 Climate 6 | Pearson Correlation | -0.041 | -0.084 | S17 Climate 6 | Pearson Correlation | -0.162 | -0.181 |
|  | Sig. (2-tailed) | 0.599 | 0.278 |  | Sig. (2-tailed) | 0.101 | 0.067 |
|  | N | 170 | 170 |  | N | 104 | 104 |
| F16 Climate 7 | Pearson Correlation | 0.074 | 0.030 | S17 Climate 7 | Pearson Correlation | 0.032 | 0.020 |
|  | Sig. (2-tailed) | 0.343 | 0.704 |  | Sig. (2-tailed) | 0.747 | 0.838 |
|  | N | 166 | 166 |  | N | 104 | 104 |
| F16 Climate 8 | Pearson Correlation | -0.102 | -0.116 | S17 Climate 8 | Pearson Correlation | -0.116 | -0.120 |
|  | Sig. (2-tailed) | 0.191 | 0.136 |  | Sig. (2-tailed) | 0.245 | 0.227 |
|  | N | 166 | 166 |  | N | 103 | 103 |
| F16 Climate 9 | Pearson Correlation | -0.069 | -0.109 | S17 Climate 9 | Pearson Correlation | 0.008 | 0.018 |
|  | Sig. (2-tailed) | 0.379 | 0.162 |  | Sig. (2-tailed) | 0.933 | 0.854 |
|  | N | 167 | 167 |  | N | 103 | 103 |
| F16 Climate 10 | Pearson Correlation | 0.027 | -0.026 | S17 Climate 10 | Pearson Correlation | -0.054 | -0.078 |
|  | Sig. (2-tailed) | 0.731 | 0.736 |  | Sig. (2-tailed) | 0.586 | 0.432 |
|  | N | 166 | 166 |  | N | 103 | 103 |
| F16 Climate 11 | Pearson Correlation | 0.033 | -0.013 | S17 Climate 11 | Pearson Correlation | -0.131 | -0.165 |
|  | Sig. (2-tailed) | 0.678 | 0.864 |  | Sig. (2-tailed) | 0.187 | 0.096 |
|  | N | 164 | 164 |  | N | 103 | 103 |
| F16 Climate 12 | Pearson Correlation | -0.106 | -0.130 | S17 Climate 12 | Pearson Correlation | -0.162 | -0.165 |
|  | Sig. (2-tailed) | 0.176 | 0.097 |  | Sig. (2-tailed) | 0.101 | 0.096 |
|  | N | 165 | 165 |  | N | 103 | 103 |
| F16 Climate13 | Pearson Correlation | -0.141 | -0.151 | S17 Climate13 | Pearson Correlation | -.215^*^ | -.269^**^ |
|  | Sig. (2-tailed) | 0.071 | 0.053 |  | Sig. (2-tailed) | 0.030 | 0.006 |
|  | N | 165 | 165 |  | N | 102 | 102 |
